# Microbial proteases and their applications

**DOI:** 10.3389/fmicb.2023.1236368

**Published:** 2023-09-14

**Authors:** Peng Song, Xue Zhang, Shuhua Wang, Wei Xu, Fei Wang, Rongzhao Fu, Feng Wei

**Affiliations:** ^1^College of Life Sciences, Liaocheng University, Liaocheng, China; ^2^Shandong Aobo Biotech Co. Ltd., Liaocheng, China; ^3^Jiangxi Zymerck Biotech Co. Ltd., Nanchang, China

**Keywords:** protease, classification, detection, expression, application

## Abstract

Proteases (proteinases or peptidases) are a class of hydrolases that cleave peptide chains in proteins. Endopeptidases are a type of protease that hydrolyze the internal peptide bonds of proteins, forming shorter peptides; exopeptidases hydrolyze the terminal peptide bonds from the C-terminal or N-terminal, forming free amino acids. Microbial proteases are a popular instrument in many industrial applications. In this review, the classification, detection, identification, and sources of microbial proteases are systematically introduced, as well as their applications in food, detergents, waste treatment, and biotechnology processes in the industry fields. In addition, recent studies on techniques used to express heterologous microbial proteases are summarized to describe the process of studying proteases. Finally, future developmental trends for microbial proteases are discussed.

## 1. Introduction

As recently highlighted by research and academic papers on enzymes, proteases constitute the largest product segment in the global industrial market for enzymes because they are extensively used in detergent and food industries (Acrofan, 2021; FOC Group, 2022). Additionally, with the development of science and technology, the use of protease enzymes in several bioremediation processes and leather treatments is increasing (Research and Markets, 2021). Moreover, protease enzymes are being extensively used in the production of medicines, as protease enzymes treat multiple diseases, such as lung, heart, eye, digestive tract, and skin ulcer diseases as well as soreness (Shrivastava et al., 2019). Thus, the demand for protease enzymes should continue to increase in the future.

The main sources of proteases are animals (e.g., calf stomach), plants (e.g., pineapple, fig, and papaya), microbes (e.g., *Bacillus* spp., *Pseudomonas* spp.; Jisha et al., 2013; Sun et al., 2016, 2019; Chitte and Chaphalkar, 2017). The production of enzymes from animal and plant sources, however, has been limited due to ethical issues, environmental reasons, and low-efficiency production processes. Commercially, microbial enzymes are popular due to their scientific and economic advantages as well as their broad biochemical diversity (Jisha et al., 2013).

In this paper, a detailed studies were reviewed on the classification, identification, testing, application and preparation of microbial protease due to their many advantages, including their rich variety (microbial proteases include acid, neutral, and alkaline proteases); ability to function under various industrial and even extreme conditions (such as high temperatures); and wide application potential and large market in various industry fields, including food, beverage, detergents, leather, animal feed, waste treatment, microbial fermentation and biotechnology industries. In addition, the number of potential proteases is very large (the main bioinformatics databases contains tens of millions of protease genes without functional verification).

## 2. Classification of microbial proteases

Microbial proteases can be categorized into the following categories: (1) proteases that can hydrolyze specific proteins (e.g., collagenase, elastase, and keratinase); (2) proteases that exhibit likeness to well-characterized proteolytic enzymes (e.g., chymotrypsin, trypsin, and pepsin); (3) proteases with an active pH range (e.g., alkaline, acid, or neutral); (4) proteases that exhibit mechanism of catalytic behavior (i.e., the amino acid residues are involved in the active site or center of the enzymes, such as aspartic proteases, cysteine proteases, metalloproteases, and serine proteases; Rao et al., 1998); and (5) proteases with hydrolysis sites specificity (endopeptidases and exopeptidases, which act internally in polypeptide chains and near the terminus of a polypeptide chain, respectively). The Enzyme Commission (EC) has denoted various endopeptidase and exopeptidase subtypes (see Table 1).

**Table 1:** Classification and nomenclature of peptidases.

Subclasses	EC code	Activity
Exopeptidases	3.4.11–19	Cleave near a terminus of peptides or proteins
Aminopeptidases	3.4.11	Remove a single amino acid from the free N-terminus
Dipeptidases	3.4.13	Exopeptidases specific for dipeptides
Dipeptidyl peptidases	3.4.14	Remove a dipeptide from the free N-terminus
Tripeptidyl peptidases	3.4.14	Remove a tripeptide from the free N-terminus
Peptidyldipeptidases	3.4.15	Release of free C-terminus liberates a dipeptide
Carboxypeptidases	3.4.16–18	Remove a single amino acid from the C-terminus
Serine proteases	3.4.16	Active sites contain serine
Metalloproteases	3.4.17	Active sites contain metal ions
Cysteine proteases	3.4.18	Active sites contain cysteine
Omega peptidases	3.4.19	Remove terminal residues that are substituted, cyclized or linked by isopeptide bonds
Endopeptidases	3.4.21–24	Cleave internally in peptides or proteins
Serine proteases	3.4.21	Active sites contain serine
Cysteine proteases	3.4.22	Active sites contain cysteine
Aspartic proteases	3.4.23	Active sites contain aspartate
Metalloproteases	3.4.24	Active sites contain metal ions
Threonine endopeptidases	3.4.25	Active sites contain threonine
Endopeptidases of unknown catalytic mechanism	3.4.99	Acting on peptide bonds

Proteases are categorized in subgroup 4 of group 3 (hydrolase), per the Nomenclature Committee of the International Union of Biochemistry and Molecular Biology (International Union of Biochemistry, 1992).

A detailed system of classification has resulted from increased knowledge on the catalytic mechanism and structure. Depending on the evolutionary relationships and amino acid sequences of proteases, they are categorized into different clans and families (Rawlings et al., 2017). A clan (i.e., a group of families) does not exhibit significant similarities in sequence but does possess an evolutionary relationship. Clans can also include families from different catalytic classes because their catalytic-site residues follow an identical order and show similar tertiary folds. A family contains proteolytic enzymes that are homologous, which is revealed by a significant similarity in their amino acid sequence. They can be identified according to the family’s enzyme type or a homologous protein to the enzyme type, which thus is a family member. Based on this classification, the MEROPS database provides comprehensive details about different proteases. According to these phylogenetic relationships and mechanisms of action, all proteases in clans and families can be grouped into asparagine proteases, aspartic proteases, cysteine proteases, glutamic proteases, mixed proteases, metalloproteases, threonine proteases, serine proteases, and unknown proteases (Rawlings et al., 2017).

## 3. Detection of microbial proteases

### 3.1. Endopeptidase detection

#### 3.1.1. Observation of halos

Protease production is indicated by the formation of clear halos around colonies that have grown on protein substrates in agar plates. This occurs when extracellular endopeptidases are produced by microorganisms in solid media. Growth media supplement the protein substrates, which were then poured into Petri plates. Commonly used substrates include skim milk agar (Masi et al., 2021; Shaikh et al., 2023), casein agar (Yokota et al., 1988; Rathod and Pathak, 2014), bovine serum albumin (BSA) agar (De Azeredo et al., 2001), gelatin agar (Mortezaei et al., 2021), keratin agar (Pereira et al., 2014; Nnolim et al., 2020), fibrin agar (Prabhu et al., 2021; Anis Ahamed et al., 2022), and elastin agar (Zins et al., 2001). When protease was produced in liquid media, the supernatant of fermentation broth (for extracellular proteases) or cell lysate (intracellular proteases) containing protease was collected. The same agar plates (containing protein substrates) as described for solid media were prepared, a well was created in the plate was made or an Oxford cup was placed on the plate for the enzyme liquid container to observe the halo (Wang et al., 2015; Yang et al., 2021).

The observation halo is the most intuitive and simple method used to identify proteases, but it is only suitable for endopeptidases and proteases that exhibit sufficiently strong activity to form clear halos. The activity of proteases is commonly detected by measuring the hydrolysate or the reduction in substrate caused by protease hydrolysis. There are many kinds of proteases that exhibit different activities, utilize different hydrolysis modes, and generate hydrolysis products with different characteristics; thus, different substrates and methods are needed to detect these proteases. To date, the substrates used to detect proteases are roughly divided into native substrates and modified substrates. The modified substrates are further mainly divided into chromogenic substrates and fluorescent substrates. Different substrates are detected with different methods.

#### 3.1.2. Detection by natural protein substrates

Natural protein substrates are those that occur in nature (plant protein, animal protein, microbial protein, etc.). The most commonly used substrate for testing protease activity is casein. Protease hydrolyzes casein under certain temperature and pH conditions to produce peptides or amino acids that are soluble in an acidic solution. After undergoing acid deposition, the newly formed product dissolves in the upper acid solution, while the unhydrolyzed protein forms a precipitate (Yokota et al., 1988; Rathod and Pathak, 2014). The supernatant is collected by centrifugation, and the activity of the protease is determined by testing the resulting peptides or amino acids using Folin reagent, ninhydrin, TNBS or OPA, which each exhibit advantages and disadvantages (Table 2).

**Table 2:** Detection of protease activity using a natural substrate.

Test method/reagent	Detection principle	Advantages and disadvantages	References
Folin reagent	Proteases hydrolyze protein substrates to produces amino acids with phenolic groups (tyrosine, tryptophan) or peptides containing amino acids with phenolic groups, which can be reduced by the Folin reagent (Folin) under alkaline conditions to produce molybdenum blue and tungsten blue, the color of which is proportional to the content of amino acids with phenolic groups. The number of amino acids with phenolic groups produced by enzymatic digestion is obtained by detecting the absorbance at 680 nm, and thus calculating the protease activity	This method is easy to operate and the quantitative range is 5–100 μg amino acids; the color reaction of Folin reagent is caused by tyrosine, tryptophan and cysteine, so if the sample contains phenols, citric acid and sulfhydryl compounds, they will interfere with the detection; This method is affected by the type of protein substrate, and the color intensity of different proteins is slightly different due to the different content of tyrosine and tryptophan	Mcdonald and Chen (1965), Chen et al. (2022)
Ninhydrin	Amino acids and peptides with free α-amino and α-carboxyl groups react with ninhydrin to produce a blue–purple substance (proline and hydroxyproline react with ninhydrin to produce a (bright) yellow substance). The color shade of this compound is proportional to the amino acid content and the amino acid content is determined by measuring the absorbance at 570 nm (440 nm for proline and hydroxyproline)	A commonly used and sensitive method for the detection of proteases with a detection limit of 0.5 μg amino acids; however, the color developer has low stability and cannot be stored for a long time; different amino acids and ninhydrin develop color differently, resulting in partial deviation of the measurement results	Moore and Stein (1948), Zhang et al. (2013), Hamed et al. (2020)
Trinitrobenzene sulfonate (TNBS)	TNBS reacted with amino acids under alkaline conditions for 1 h at 37°C and cooled at room temperature for 30 min, followed by the detection of absorbance values at 420 nm, which were proportional to the amino acid content	The sensitivity of this method is reasonable, and the detection range is 0.05–0.4 μmol amino acids; the shortcomings are that the assay is time-consuming, the ε-amino group of leucine can also react with TNBS, which affects the accuracy of the determination results, and the lack of correlation between the proline and hydroxyproline contents and the absorbance values, which can easily produce bias	Adler-Nissen (1979), Spellman et al. (2003), Fathi et al. (2021), Han et al. (2021), Cermeño et al. (2022)
o-phthaldialdehyde (OPA)	Proteases release a free amino group for each peptide bond hydrolyzed. The free amino group reacts with OPA to form a yellow complex, the absorbance of which can be measured spectrophotometrically at 340 nm	The OPA method is a rapid and simple method to determine the hydrolysis of protein hydrolysates, and is 5–10 times more sensitive than the ninhydrin method; OPA determination of hydrolysis relies on a weak and unstable reaction between OPA and cysteine in the hydrolyzed substrate; this method is not suitable for cysteine-rich substrates, and proline and OPA do not react and cannot be detected; in addition, the detection process requires strict time control, because the detection value changes with time, and it is relatively difficult to obtain accurate measurement values	Church et al. (1983), Spellman et al. (2003), Hong et al. (2022), Alblooshi et al. (2023)

#### 3.1.3. Detection by modified protein substrates

##### 3.1.3.1. Detection by chromogenic substrates

To increase substrate solubility and detection sensitivity, modified protein substrate is used in some methods to detect protease activity, and this substrate should generate a colored end product after proteolysis or a product that can be converted into a colored complex. One example is azocasein, a casein dyed with *p*-aminobenzenesulfonic acid, which produces a colored complex that is soluble in trichloroacetic acid and shows absorption at 440 nm after digestion by proteases (Cejudo-Bastante et al., 2022; de Matos et al., 2022; Marson et al., 2022). Succinyl casein, which possesses chemically succinlyated amino groups (Hatakeyama et al., 1992), easily dissolves at pH values greater than 4, unlike casein.

According to substrate specificity, the synthetic substrate can be identified by the type of protease screened, such as Tosyl-Gly-Pro-Arg-*p*NA for trypsin (Sandholt et al., 2018), Suc-Ala-Ala-Pro-Phe-*p*NA for chymotrypsin (Siigur et al., 2011; Németh et al., 2022) and Suc-Ala-Ala-Pro-Val-*p*NA for elastase (Ferreira et al., 2009). However, N-Cbz-Ala-Ala-Leu-*p*NA and N-Cbz-Gly-Gly-Leu-*p*NA are good substrates for subtilisins (Burchacka et al., 2022). The principle underlying the assay is that proteases hydrolyze the amide bond connecting *p*-nitroaniline (*p*NA) to the neighboring amino acid residue, and released *p*NA exhibits specific absorption at a 405 nm wavelength (enzyme activity is proportional to fluorescence intensity).

##### 3.1.3.2. Detection by fluorogenic substrates

More sensitive methods are needed when the quantity or activity of protease enzymes are low, and sensitive fluorescent peptide substrates are available, through which the limit of detection reaches the ng level (Austin et al., 2022).

Fluorescent labeling applied to protease substrate modification can be divided into the following categories: 1, single fluorescence-based labeling, in which one kind of fluorescent dye labels the substrate protein after binding so that the substrate protein obtains fluorescent labeling. 2, Double fluorescence labeling, in which two different fluorescent dyes label the peptides. One dye is an energy acceptor and the other is an energy donor; the labeled peptide, which is activated by protease hydrolysis, does not show fluorescence. 3, Homotransfer fluorescence labeling, in which there is one kind of fluorescence labeling substrate protein, and fluorescence resonance energy transfer (FRET) occurs between the labeled fluorescent molecules, which do not show fluorescence but are hydrolyzed by proteases to activate fluorescence.

###### 3.1.3.2.1. Single fluorescence-based labeling

Single fluorescence dye labeling involves introducing fluorophores attached to side chain amino acids, such as the N-terminus, C-terminus, Glu, Lys or Cys of a peptide. Nearly 30 types of fluorescence dyes have been developed thus far (Díaz-García and Badía-Laíño, 2018). The more widely used dyes are carboxyfluorescein (FAM; Feng et al., 2022), fluorescein isothiocyanate (FITC; Taylor et al., 2022), dansyl chloride (DNS-Cl; Yoo and Han, 2021), 2,4-dinitrophenylhydrazine (Dnp; Oliveira et al., 2001), 7-amino-4-methylcoumarin (AMC), 7-amino-4-trifluoromethyl coumarin (AFC; Breidenbach et al., 2020), carboxyrhodamine 110 (CR110; Lorey, 2002), Texas Red (Lorey, 2002), pentamethine cyanine (Cy5) and heptamethine cyanine (Cy7) dyes (Chin and Kim, 2018). Protease activity is measured as an enhanced emission generated after a peptide is cleaved by an enzyme and is released from the fluorophore. The detection limits of single fluorescence-based labeling for proteases can reach the ng level (Kasana et al., 2011). However, when detection is performed using a single fluorescently labeled protease substrate, the product and substrate must be separated, and the pH needs to be adjusted to enhance the detection signal. The detection steps remain relatively complex (Twining, 1984; Austin et al., 2022).

###### 3.1.3.2.2. Fluorescence dye double labeling

In contrast to single fluorescence-based labeling, such as the commonly used FTC-casein assay, double fluorescence labeling provides a more convenient and precise method, which is based on the FRET concept (Clapp et al., 2004; Goulet et al., 2020). The kinetics of exo- and endopeptidases can be measured over a wide pH range using assay procedures that do not involve separation steps (Legare et al., 2022). The total substrate turnover can be measured at a fixed time after an enzyme is added (Elston et al., 2007). Decreased fluorescence quenching (i.e., increased total fluorescence), which occurs as peptides (labeled proteins) are digested into smaller fluorescein-labeled fragments, can be identified using FRET-based measurement. In classical FRET, electron energy transfer occurs between two fluorophores, an energy acceptor and energy donor. Table 3 lists common combinations of acceptors and donors.

**Table 3:** Double fluorescence labeled donor-acceptor pair.

Acceptors	Donors	Wavelength (nm)	
		Excitation	Emission
Dnp (2,4-Dinitrophenyl)	Trp (Tryptophan)	280 nm	360 nm
4-Nitro-Z (4-Nitro-benzyloxycarbonyl)	Trp (Tryptophan)	280 nm	360 nm
Dnp (2,4-Dinitrophenyl)	Mca (7-Methoxycoumarin-4-acetyl)	325 nm	392 nm
pNA (para-Nitroaniline)	Abz (2-Aminobenzoyl)	320 nm	420 nm
3-Nitro-Tyr (3-Nitro-tyrosine)	Abz (2-Aminobenzoyl)	320 nm	420 nm
4-Nitro-Phe (4-Nitro-phenylalanine)	Abz (2-Aminobenzoyl)	320 nm	420 nm
Dabcyl ((4-(4-Dimethylamino)phenyl)azo)benzoyl	EDANS (5-[(2-Aminoethyl)ami-no]-1-naphthalenesulfo-nic acid)	340 nm	490 nm
Dabsyl (4-(4-Diethylaminophenylazo)-benzenesulfonyl)	Lucifer Yellow	430 nm	520 nm
Dnp (2,4-Dinitrophenyl)	FITC (Fluorescein isothiocyanate)	490 nm	520 nm
4-Nitro-Phe (4-Nitro-phenylalanine)	Dansyl (5-(Dimethylamino) naphthalene-1-sulfonyl)	342 nm	562 nm
QSY7	5-TAMRA (Carboxytetramethyl rhodamine)	547 nm	573 nm
QSY-7	Eu (III) Chelate	340 nm	613 nm

###### 3.1.3.2.3. Homotransfer fluorescence labeling

**Figure 1:**
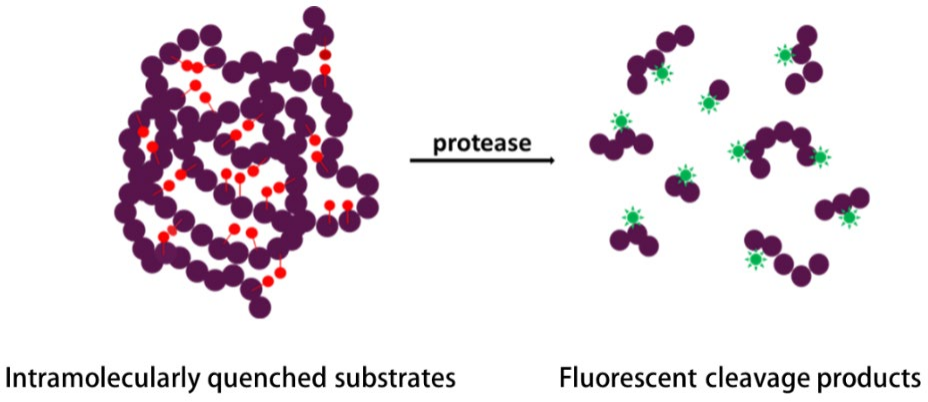
Principle of protease detection by fluorescence homotransfer.

As mentioned above, classical FRET involves electron energy transfer between two different fluorophores; however, FRET events can also occur as a result of fluorescence homotransfer in which fluorescein acts both as the energy “donor” and energy “acceptor” (Runnels and Scarlata, 1995; Chen et al., 2000; Thompson et al., 2000; Figure 1), which is called homotransfer fluorescence labeling. Compared to the FTC-casein assay, these assays are also easier to perform, and they are 100-fold more sensitive (Jones et al., 1997).

A typical single-fluorescence dye used in FRET is the BODIPY dye (Jones et al., 1997): BODIPY dye molecules are attached to casein to prepare casein conjugates of BODIPY dyes. The dyes in these conjugates are labeled to achieve efficient quenching in the protein. This process yields nonfluorescent substrate molecules. These fluorogenic substrates release highly fluorescent BODIPY dye-labeled peptides during proteolysis and increase the fluorescence as it relates to enzymatic activity. Using standard fluorometers, filter fluorometers, or fluorescence microplate readers, this activity can be measured. Fluorescein excitation and emission wavelengths can be used to measure BODIPY casein hydrolysis. EnzChek™ Protease Assay kits from ThermoFisher Scientific contain a heavily labeled casein derivative. Green-fluorescent BODIPY FL dye and red-fluorescent BODIPY TR-X dye are commonly used for this application.

### 3.2. Exopeptidase detection

Endopeptidases hydrolyze proteins and mainly release peptides, and exopeptidases hydrolyze proteins and release free amino acids, so the methods used to detect endo−/exopeptidases must be different, and the method used to detect endopeptidases is not very sensitive to exopeptidases.

The assay used to measure exopeptidase activity usually involves synthetic peptide as the substrate; for the aminopeptidase assay, a peptide with two and three amino acid residues is synthesized to detect aminopeptidases (Mathew et al., 2000; Gu and Walling, 2002). For more sensitive detection, *p*-nitroaniline (*p*NA; Cahan et al., 2001; Schulze et al., 2018) or 7-methoxycoumarin-4-acetic acid (MCA; Chen et al., 2011, 2012; Schulze et al., 2018) are connected to the carboxyl terminus of peptides; after hydrolysis, a free *p*NA or MCA molecule is released in the reaction solution. This method can detect the specific absorbance value to determine the aminopeptidases activity.

For the carboxypeptidase assay, a peptide with an amino terminus blocked by benzyloxycarbonyl (CBZ; Fu et al., 2011; Song et al., 2021a) or benzoyl (BZ; Ramirez Zavala et al., 2004; Heylen et al., 2010) is most commonly used as a substrate. Only carboxypeptidase can release amino acids from the carboxyl terminus. After hydrolysis, free amino acids are released from the synthetic peptide and detected by ninhydrin or OPA reagent.

Fluorescent substrates can also be used to detect exopeptidases, including aminopeptidases (Chen, 2020; Liu S. Y. et al., 2022; Ma et al., 2023) and carboxypeptidases (Xiong et al., 2018; Yoo and Han, 2021), because of their extreme sensitivity.

Some protease detection methods, such as ELISAs or ultrasonic resolver technology assays, are also available. These methods are not widely used due to their limitations and are only used in special cases. For example, prior information on the structure of the enzyme is needed to perform ELISA-based assays (Blair and McDowell, 1995). For ultrasonic resolver technology, a different analytical method is needed and must be first correlated to the corresponding ultrasonic velocity signals in advance (Born et al., 2010). These methods will not be introduced in detail here. For details, please refer to related reviews (Kasana et al., 2011).

Among microbial resources, potential proteases are extremely abundant, and proteases detection methods are crucial for developing novel proteases. In the future, detection methods will be developed that are sensitive, fast, inexpensive, and suitable for high-throughput screening of proteases.

## 4. Application of microbial proteases

**Figure 2:**
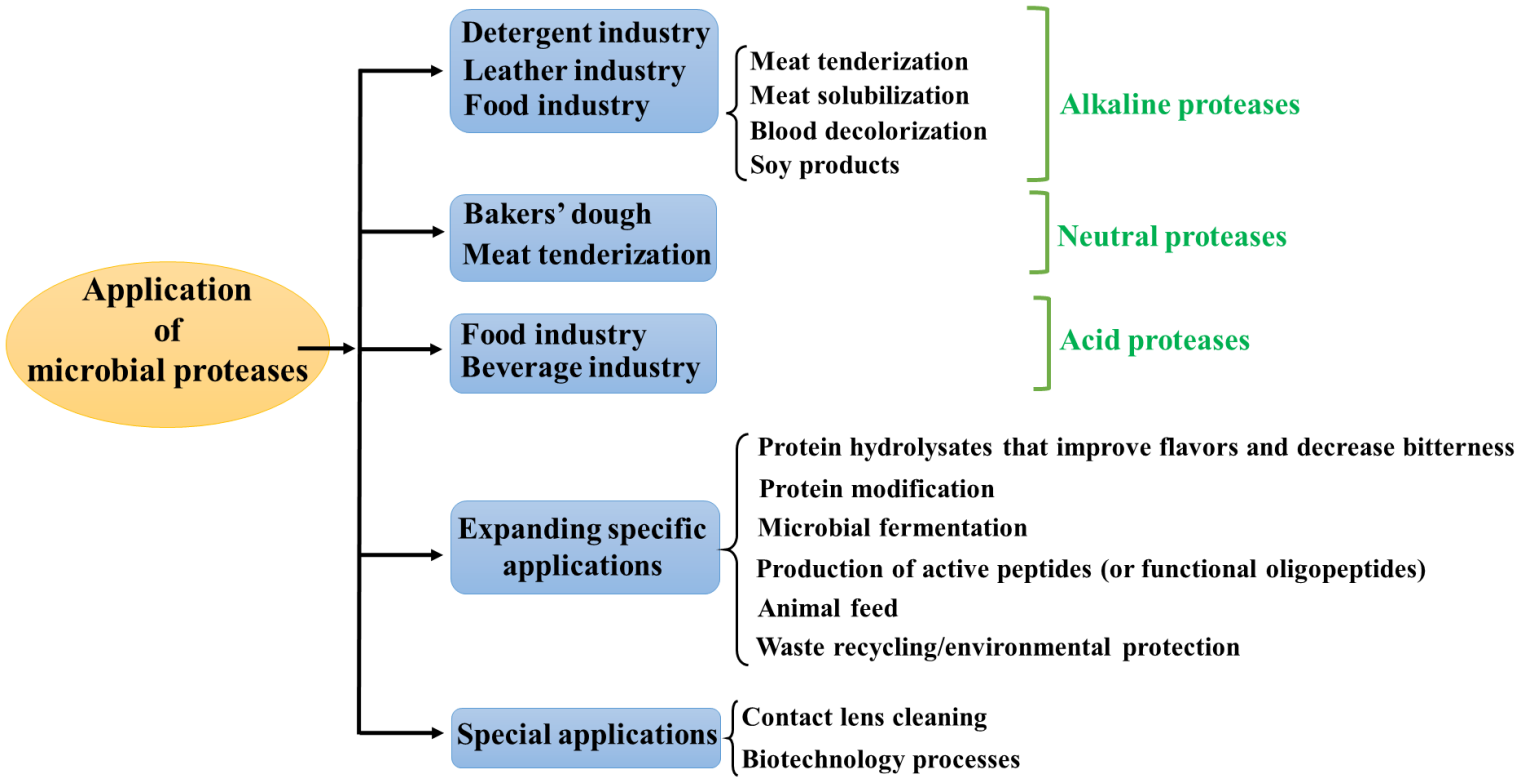
The applications of proteases.

Microbial proteases have wide ranging applications in several fields, including baking, brewing, detergents, leather making, pharmaceuticals, meat tenderizing, cosmetics, medical diagnosis and so on (Christensen et al., 2022; Reddy et al., 2022; Akram et al., 2023; Mubeen et al., 2023). In addition, with the rapid development of new fields, applications of microbial proteases are expanding to new areas, such feed industries (Bernardeau et al., 2022; Cupi et al., 2022), hydrolysis applications to prepare active peptides (Christensen et al., 2022), and environmental protection applications, such as waste treatment and reuse (Ariaeenejad et al., 2022; Asitok et al., 2022; Zhai et al., 2022). These applications illustrate the diversity and importance of proteases. The applications of proteases and their respective microbial sources by examining acid protease, neutral protease and alkaline proteases and their classification were discussed and briefly summarized in Figure 2.

### 4.1. Alkaline proteases

Among the different proteases, alkaline proteases exhibit the highest activity in the pH range of 8 to 13. Alkaline proteases are commonly used in the following industries:

#### 4.1.1. Detergent industry

Alkaline proteases represent the largest share of the enzyme market, are a commercially important group of enzymes and are used primarily as detergent additives (Sharma et al., 2017). By adding alkaline proteases to laundry detergents, proteinaceous material can be released from stains (Matkawala et al., 2019; Tanwar et al., 2022). Unlike traditional detergents, the addition of protease saves energy and improves washing efficiency. After soaking, shorter periods of agitation and lower wash temperatures can be used with the addition of proteases (Mubeen et al., 2023). Commercial alkaline proteases are effective at low levels (0.4–0.8%) and are compatible with various detergent components that contain oxidizing and sequestering agents. These proteases also exhibit high activity and stability over a broad range of pH values and temperatures as well as a long shelf life (Vojcic et al., 2015). Proteases are environmentally friendly, nonphosphate detergents, and washing powders containing proteases can be used in dry cleaning applications as stain and spot removers (Kumar et al., 2016).

#### 4.1.2. Leather industry

Leathers are usually processed using an alkaline reagent. Because alkaline proteases exhibit keratinolytic and elastolytic activities, they can effectively biotreat leather, particularly the bating and dehairing of hides and skins (Tian et al., 2019; Srivastava et al., 2020). These methods are better choices than conventional methods, which use harsh chemicals, create disposal problems, exhibit increase safety risks, and cause chemical pollution (Hassan et al., 2020). Subsequent studies have successfully used alkaline proteases from *Aspergillus*, *Streptomyces*, and *Bacillus* in leather tanning (Ogino et al., 2008; Paul et al., 2016; El-Ghonemy and Ali, 2021; Hasan et al., 2022; Zhang et al., 2022).

#### 4.1.3. Food industry

The most extensive application of alkaline protease is in the food industry.

##### 4.1.3.1. Meat tenderization

Alkaline proteases can hydrolyze muscle fiber proteins and connective tissue proteins. Meat tenderization is achieved by immersing meat in a protease solution or sprinkling it with a powdered enzyme (Bureros et al., 2020). The vascular systems of animals are often injected with protease solutions 10–30 min before slaughter (Kalisz, 1988), including alkaline elastase (Qihe et al., 2006) and thermophilic alkaline protease (Wilson et al., 1992).

##### 4.1.3.2. Meat solubilization

Soluble meat hydrolysates and meat-flavored hydrolysates are byproducts of the leather industry. These potential sources of protein are bone, offal (raw lung), and bone residues after mechanical deboning. The most beneficial enzyme in terms of solubilization, cost, and other factors is alcalase (Anzani et al., 2017), which can be used to produce fish protein hydrolysates (Noman et al., 2022).

##### 4.1.3.3. Blood decolorization

Because of its intense color, blood is an underutilized source of food protein. Although the red cell fraction contains 75% of the protein in the blood, alcalase is preferred because it thoroughly and rapidly hydrolyzes red cells.

The red cell fraction contains 75% of the protein in the blood, of which more than 92% is hemoglobin. Hemoglobin is composed of heme and globin, and heme causes blood products to eventually appear black red and exhibits a strong bloody smell. Alcalase is the preferred blood decolorization protease because it thoroughly and rapidly hydrolyzes hemoglobin and releases polypeptides. After enzymatic cleavage, the remaining hydrophobic core formed by wrapping heme with hydrophobic peptide fragments forms precipitates under appropriate pH conditions. The supernatant is dried by spray to produce hemoglobin powder, which can remove the ugly black purple color and the bloody smell of blood products; furthermore, the powder can be used as feed additive, colorants in the food industry and pharmaceutical raw materials in the pharmaceutical industry (Pérez-Gálvez et al., 2011).

##### 4.1.3.4. Soy products

In Asia, fungal proteases have long been used to prepare soy sauce and soy products (Devanthi and Gkatzionis, 2019). The alkaline and neutral proteases of *Aspergillus* are essential in the digestion of soybean protein and provide the rich flavor of true soy sauce (Zhao et al., 2020). They also play an important role in improving the quality of soy products during processing (Xu et al., 2013).

Members of the genus *Bacillus* have been screened for use in various industrial applications and have been identified as the predominant alkalophilic microorganism. They are a prolific source of alkaline proteases, including *Bacillus amyloliquefaciens*, *Bacillus licheniformis*, and some *Bacillus* sp. Many fungi produce extracellular alkaline proteases, most notably *Aspergillus* sp. (Table 4).

**Table 4:** Representative alkaline proteases originated from microbial sources.

Microbial sources	Proteases	References
*Bacillus amyloliquefaciens*	An extracellular alkaline protease	Chen W. et al. (2022)
	An alkaline proteases AprM	Xie et al. (2022)
	An alkaline serine-protease APR68	Cho (2019)
*Bacillus licheniformis*	An alkaline protease AprE	Zhou et al. (2021)
	A detergent stable thermophilic alkaline protease	Emran et al. (2020)
	Asubtilisin protease	Cupi et al. (2022)
*Bacillus* sp.	An Alkaline protease with special reference to contact lens cleansing	Rejisha and Murugan (2021)
	A novel alkaline serine protease	Ariyaei et al. (2019)
	A H_2_O_2_-tolerant alkaline protease	Yu et al. (2019)
Deep-sea fungi	An alkaline and cold-tolerant proteases	Damare et al. (2006)
*Aspergillus oryzae*, *Penicillium roquefortii* and *Aspergillus flavipes*	Several alkaline proteases	Novelli et al. (2016)
*Aspergillus* sp.	An activator-dependent protease	Zhu X. et al. (2021)
	An alkaline protease	Salihi et al. (2017)
	An alkaline serine protease	Yadav et al. (2015)
	An extracellular keratinolytic protease	Anitha and Palanivelu (2013)
	A solvent, salt and alkali-tolerant alkaline protease	Abdel Wahab and Ahmed (2018)

### 4.2. Neutral proteases

Neutral proteases exhibit the highest activity at neutral, weakly alkaline or weakly acidic pH values. Neutral proteases are used in the following applications.

#### 4.2.1. Bakers’ dough

To help in bread production, neutral proteases and amylases can be added to wheat or flour. Protease increases bread volume, improves dough elasticity, and improves crust texture (Zadeike et al., 2018; Gu et al., 2022; Xu et al., 2022; Li J. et al., 2023; Sun et al., 2023). In the process of making crackers, biscuits, and cookies, neutral proteases are used to improve the extensibility and strength of the dough and prevent dough from tearing when rolled thin. To prevent biscuits from bending and wrinkling in the oven, the dough must be soft (Borrelli et al., 2003; Sumantha et al., 2006; Mokashe et al., 2018; Nikinmaa et al., 2019). A soft and pliable dough is also necessary for the precise letters and decoration on biscuits. Bacterial neutral proteases are often used to achieve this (Ehren et al., 2009) because the enzymes’ highly specific endopeptidases are ideal for high protein flours.

#### 4.2.2. Meat tenderization

Fresh meat pH is neutral, and therefore neutral proteases are best suited for hydrolysis; tenderization of meat is achieved by the action of endogenous proteases, especially neutral lysosomal cathepsins and neutral metalloprotease/cysteine endopeptidase (Prates et al., 2001; Thomas et al., 2004; Mikołajczak et al., 2019).

Neutral proteases are widely distributed among the *Bacillus* and *Aspergillus* species (Ward et al., 2009). Thermolysin [EC 3.4.24.27], which is produced by *Bacillus thermoproteolyticus*, is probably the best-known neutral protease (Inouye et al., 2007). Thermolysin was originally identified in the culture broth of *Bacillus thermoproteolyticus* Rokko and is an attractive target in protein engineering. Since its discovery in 1962, Thermolysin, which is a thermostable neutral zinc metalloprotease, has undergone extensive structural and mechanistic studies due to its halophilicity, catalytic mechanism, and thermostability. The *Bacillus* genera that produce neutral proteases include *Bacillus subtilis*, *Bacillus licheniformis*, *Bacillus stearothermophilus*, *Bacillus nakamurai*, and *Bacillus tropicus*, and the *Aspergillus* genera include *Aspergillus oryzae*, *Aspergillus niger*, *Aspergillus sojae*, *Aspergillus nidulans*, and *Aspergillus tamarii* (Table 5).

**Table 5:** Representative neutral proteases originated from microbial sources.

Microbial sources	Proteases	References
*Bacillus subtilis*	A neutral protease	Albillos et al. (2007)
*Bacillus licheniformis*	Neutral proteases	Reddy et al. (2022)
*Bacillus stearothermophilus*	A neutral protease	Mansfeld et al. (2005)
	A neutral protease	Eijsink et al. (1991)
*Bacillus nakamurai*	An Extracellular protease	Shaikh et al. (2023)
*Bacillus tropicus*	Keratinolytic proteases	Liya et al. (2023)
*Aspergillus oryzae*	A thermolysin-like protease, neutral protease I	Ma et al. (2016)
	Neutral proteases	Belmessikh et al. (2013)
*Aspergillus niger*	Neutral proteases	Reddy et al. (2022)
*Aspergillus sojae*	Neutral proteases	Li et al. (2023)
*Aspergillus nidulans*	Neutral proteases	Campbell and Peberdy (1983)
*Aspergillus tamarii*	Neutral proteases	Silva et al. (2018)

### 4.3. Acid proteases

The proteases described here are active between pH 2 and 6. Acid proteases of microbial origin are mostly found in the food and beverage industries.

#### 4.3.1. Food industry

Acid proteases are primarily used in the food industry for the clotting of milk during the manufacturing of cheese. When the milk proteins coagulate, they form solid masses or curds. Then, the whey is removed to generate cheese (Tsuchiya et al., 1993; Hellmuth, 2006; Theron and Divol, 2014). In addition to their application in the dairy industry, acid proteases are also used for baking. Similar to neutral proteases, acid proteases from *Aspergillus oryzae* can limit the proteolysis of wheat gluten and increase loaf volume. Fungal-derived acid proteases have also been extensively applied to create food seasonings and improve protein-rich foods (e.g., bread and related foodstuffs; Hamada et al., 2013; Purushothaman et al., 2019; Wu et al., 2022; Li X. et al., 2023; Niu et al., 2023).

#### 4.3.2. Beverage industry

Acid proteases can degrade proteins in fruit juices and certain alcoholic beverages that cause turbidity (Espejo, 2021; Pati and Samantaray, 2022; Rasaq et al., 2023), including black currant (Landbo et al., 2006); cherry (Pinelo et al., 2010); pomegranate (Cerreti et al., 2017); and apple, orange, grape, and kiwi fruit juices (Guo et al., 2019). By adding acid proteases, the immediate turbidity is significantly reduced. Adding proline-specific proteases from *Aspergillus niger* (Lopez and Edens, 2005) or *Aspergillus oryzae* (Kang et al., 2014) when brewing beer can prevent chill-haze formation. This result indicates that proline-rich proteins perform hydrolysis due to a peptide fraction that cannot interact with polyphenols. Protein haze is also a problem that occurs during the production of white wine. Early research has found that by using acid proteases in wine, protein haze formation can be reduced without damaging wine quality (Marangon et al., 2012; Van Sluyter et al., 2013; Theron et al., 2018). Apart from preventing protein haze, acid proteases also increase the α-amino nitrogen concentration necessary for microbial growth and generate better flavor during beer brewing (Bell and Henschke, 2005; Lei et al., 2013; Wang et al., 2013; Serna-Saldivar and Rubio-Flores, 2017).

Acid proteases are mainly aspartic proteases and are distributed across all forms of life, including vertebrates, plants, fungi, bacteria and viruses (Theron and Divol, 2014). However, fungus-derived acid proteases, such as Aspergillopepsins I and II from *Aspergillus niger* are most commonly used in the food and beverage industries (Ichishima, 2004; Takahashi, 2004). They are the first and most commonly used acid proteases in the food industry. Recent reports on fungi-derived acid proteases have been used for various purposes, and the proteases mainly originate from *Aspergillus oryzae*, *Aspergillus niger*, *Aspergillus foetidus*, *Aspergillus saitoi*, *Aspergillus clavatus*, *Rhizomucor miehei*, *Mucor miehei*, and *Rhizopus rhizopodiformis* (Table 6).

**Table 6:** Representative acid proteases originated from microbial sources.

Microbial sources	Proteases	References
*Aspergillus oryzae*	An aspartate protease	Vishwanatha et al. (2009)
	An acid protease as starter culture in doubanjiang fermentation	Niu et al. (2023)
	A valuable food acid protease	Murthy and Kusumoto (2015)
	An acid protease	de Castro and Sato (2014)
	An aspartate protease	Shu et al. (2020)
	A salt-tolerant acid protease	Lee et al. (2010)
*Aspergillus niger*	Acid proteinase A	Takahashi et al. (1998)
	Extracellular acid proteases	Aalbæk et al. (2002)
	Acid proteinases	Yang and Lin (1998)
	Aspartic proteases	Purushothaman et al. (2019)
	Acid proteinases	Li et al. (2020)
*Aspergillus foetidus*	An aspartic protease	Souza et al. (2017)
	A thermostable extracellular acid protease	Souza et al. (2015)
*Aspergillus saitoi*	An acid carboxypeptidase	Chiba et al. (1993)
*Aspergillus clavatus*	An extracellular acid protease	Sampaio Silva et al. (2011)
*Rhizomucor miehei*	Acid proteinases	Aljammas et al. (2022a,b)
	Rennet	Celebi et al. (2016)
	Rennet	Soltani et al. (2019)
*Mucor miehei*	Acid proteases	Ayhan et al. (2001)
	Acid proteases	Escobar and Barnett (1995)
	Rennet	Seker et al. (1999)
*Rhizopus rhizopodiformis*	Extracellular acid proteases	Schindler et al. (1983), Sun et al. (2014)

### 4.4. Expanding specific applications

Protease applications are still expanding as specific applications develop, and new areas of interest in recent years are described in the following section.

#### 4.4.1. Protein hydrolysates that improve flavors and decrease bitterness

Due to their amino acid sequence and length, oligopeptides exhibit different flavors, including sweet, bitter, umami, sour, or salty taste. Twenty common amino acids also present different flavors, such as umami, sweetness and bitterness; glutamic acid presents an umami flavor; arginine, proline, leucine, isoleucine, phenylalanine, and tryptophan present a bitter taste for humans; and L-alanine and L-serine provide a sweet taste. Proteases (mainly endopeptidases) can hydrolyze proteins to produce oligopeptides, thus enhancing the flavor of protein-based food (Wang H. et al., 2022; Yan et al., 2022), and exopeptidases (mainly aminopeptidases and carboxypeptidases) can hydrolyze peptides to produce free amino acids, also enhancing (enriching) the flavor of protein-based food (Cheung et al., 2015; Fu et al., 2020; Ding et al., 2022). For example, Alcalase and Flavorzymes were used to prepare defatted flaxseed meal protein hydrolysates (Wei et al., 2018). After processing optimization, peptides with molecular weights above 1,000 Da enhanced the texture of food, while peptides with molecular weights ranging from 128 to 1,000 Da provided meat-like flavors and influenced other sensory features.

Aminopeptidases from *Lactobacillus casei*, *Lactobacillus curvatus*, and *Lactobacillus sake* were used to improve the sensory quality of dry fermented sausages (Nandan and Nampoothiri, 2020). Neutrase, which is a neutral bacterial protease, can modify flavor in dairy applications (Sumantha et al., 2006). During the fermenting of fish sauce, taste formation is affected by protease activity because it alters the content of Ala, Asp, Glu, Leu, Lys, TCA-soluble peptides, and succinic acid (Zhu W. et al., 2021).

Bitterness is inevitably produced when oligopeptides undergo protein hydrolysis, and the intensity of bitterness of hydrolysis products is mainly related to the content and position of hydrophobic amino acids (or more accurately, amino acid residues with *Q* values above 1,500 cal/mol, such as Leu, Ile, Phe, Tyr, Trp, Pro, Val, and Lys (Nishiwaki et al., 2002) in the peptide segment). Matoba and Hata (1972) described in detail that the bitterness of protein hydrolysate is great when hydrophobic amino acids are internal in the oligopeptides, the bitterness is comparatively weaker when the hydrophobic amino acid(s) are located at either the N- or C-terminus and the weakest occurs when the hydrophobic amino acids are in the free state. Therefore, specific endopeptidases and exopeptidases can reduce the bitterness of protein hydrolysate (FitzGerald and O'Cuinn, 2006; Soeryapranata et al., 2007). Endopeptidases can hydrolyze the hydrophobic amino acids forming bonds of oligopeptides and reduce bitterness (Capiralla et al., 2002; Edens et al., 2005; Zhang M. et al., 2021). Aminopeptidases (Lin et al., 2020; Nandan and Nampoothiri, 2020; Song et al., 2020a; Nakamura et al., 2023; Wang et al., 2023) and carboxypeptidase (Ding et al., 2022) from many different sources can continue to hydrolyze end hydrophobic amino acids and then reduce bitterness.

#### 4.4.2. Protein modification

Microbial proteases are used to modify proteins. Protease-limited enzymatic hydrolysis of soybean protein can improve its solubility, emulsification, foaming and digestibility. Hydrolysis of peanut protein concentrates with *Aspergillus oryzae* crude protease extract resulted in their higher water- and oil-binding capacity as well as improved solubility, foam stability, and foaming capacity (Yadav et al., 2022). When soybean protein isolate (SPI) was treated with alkaline protease accompanied by high-speed shearing homogenization, it significantly improved the emulsion stability of the SPI hydrolysates. As a result, the foaming properties of SPI were improved significantly (Hao et al., 2022). Recent studies have examined methods to use microbial proteases from a variety of sources to improve the chemical and physical properties of animal (Ai et al., 2019; Du et al., 2022) and plant proteins (Zhang Q. et al., 2021; Lin et al., 2022; Liu Y. Q. et al., 2022; Ren and Li, 2022; Wang T. et al., 2022; Hariharan et al., 2023; Lv et al., 2023; Vogelsang-O’Dwyer et al., 2023).

#### 4.4.3. Microbial fermentation

Proteases can hydrolyze the protein substrate in the fermentation medium into small peptides, making it easier for microorganisms to quickly absorb and utilize these substrates, improving fermentation efficiency. Other studies have found that during synergistic fermentation of bean dregs and soybean meal, adding multiple strains and protease promotes strain growth, organic acid secretion and amylase secretion and reduces sugar metabolism (Heng et al., 2022). Producing ethanol by microbial fermentation will cause hydrolysis by endogenous proteases and as a result will generate amino acids and peptides. Amino acids and peptides can support the growth of microorganisms, which subsequently increases ethanol production. To improve ethanol yield and reduce fermentation time, exogenous proteases can be used to hydrolyze protein sources available in the raw materials in feedstock used for ethanol production (Thomas and Ingledew, 1990). During high-gravity ethanol production from rice, proteases increased ethanol yield and decreased fermentation time during no-cook processes. Proteases have a significant impact on the size and growth of yeast and were found to enhance ethanol content by 2.4% v/v and shorten fermentation time by 48 h. External nitrogen addition was not needed for the SLSF-VHG process of rice (Tien et al., 2022).

#### 4.4.4. Production of active peptides (or functional oligopeptides)

Active peptides are oligopeptides with specific compositions and sequences of amino acids. They are found in plant and animal proteins, and proteases can specifically hydrolyze proteins and release active peptides. Antioxidative, antidiabetic, antihypertensive, antimicrobial, antitumor, hypocholesterolemic, and many other biological properties may benefit from bioactive peptide structures (Karami and Akbari-adergani, 2019; Mada et al., 2019).

Microbial proteases have been used to produce high-value protein hydrolysates (Tacias-Pascacio et al., 2020; Mirzapour-Kouhdasht et al., 2021), especially antioxidant peptides (Mukhia et al., 2021; Noman et al., 2022; Pan et al., 2022). These proteases can be used in health food and cosmetic fields and show great application potential.

#### 4.4.5. Animal feed

Processing feed ingredients and applying exogenous proteases are the primary uses of exogenous proteases in animal feed. These proteases can be used to maintain high performance and reduce dietary protein levels. Enzymatic hydrolysis is the best method when processing animal byproducts or plant-source feedstuffs. Interesting activities from peptides from plant or animal sources include antihypertensive, antimicrobial, antioxidant, and immunomodulatory activities. The environment also benefits from proteases by improving the utilization of protein materials and reducing nitrogen and ammonia excretions (Philipps-Wiemann, 2018; Hejdysz et al., 2020).

Proteases are used in the following applications:

##### 4.4.5.1. Livestock feed

Adding *Bacillus licheniformis* to nursery diets that contain a low protein level can significantly improve nutrient digestibility, growth performance, and intestinal morphology of weaned pigs (Park et al., 2020). Keratinolytic proteases can also use low-energy consumption to convert poultry feathers to a nutritionally upgraded protein-rich feedstuff for livestock from a potent pollutant (Onifade et al., 1998).

##### 4.4.5.2. Poultry feed

Protease supplementation can improve the growth performance of broilers. HuPro protease can be supplemented under low-protein conditions to achieve a breeding effect that is similar to a positive control (antibiotic). Proteases can alter the bacterial diversity in the cecum, which has a positive effect on broilers (Wang Y. et al., 2022).

##### 4.4.5.3. Aquafeed

To improve the juiciness, flavor, tenderness, healthiness, and antioxidant capacity of grass carp meat, soy protein hydrolyzed by proteases has been added to a low-protein diet (Song et al., 2020b).

#### 4.4.6. Waste recycling/environmental protection

To process various forms of protein-rich waste, proteases can be used for liquid, solid, and hazardous waste.

Tannery wastewater microbiota was screened for metagenome-derived PersiProtease1. The novel PersiProtease1 was extracted from the microbiota and was applied to biodegrade tannery wastewater protein, dehairing sheepskins, whey protein, chicken feathers, and waste X-ray films (Ariaeenejad et al., 2022).

Several studies have found that proteases exhibit excellent deproteinization for chitin processing of shrimp waste (Jellouli et al., 2011; Mhamdi et al., 2017a,b; Doan et al., 2019).

Another promising pathway for economic benefits and reduced carbon emissions in waste-activated sludge management is the recovery of short-chain fatty acids through anaerobic fermentation. Through alkaline protease–based pretreatment, waste-activated sludge flocs can be disintegrated following cell lysis, which releases biodegradable organic matter. This approach increased the α-glucosidase activities and endogenous protease, facilitated the biodegradation of dissolved organic matter, and encouraged short-chain fatty acid production. This is a promising method for disposing waste-activated sludge and recovering carbon. Short-chain fatty acids might meet 60% of the carbon gap in wastewater, making it a cost-effective and carbon-beneficial technology to manage (Pang et al., 2020, 2022).

Efficient waste-activated sludge dewatering can be achieved through neutral protease. Waste-activated sludge treatment and disposal in wastewater treatment plants require sludge dewaterability. After enzyme conditioning, the sludge supernatant of polysaccharides, proteins, and SCOD content increased, which demonstrated the excellent performance of neutral protease. The capillary suction time increased, and the sludge water content decreased (Kang et al., 2023).

Skatole, the main source of foul odor from feces, is released from the cecum and colon of pigs and is the main source of air pollution in the pig farming environment. A new protease from *Lactobacillus brevis* has been used to remove odor from pig manure (Meng et al., 2013).

Approximately 5–7% of the total weight of chicken originates from features, which are a major pollutant because of their recalcitrant nature. Feathers are composed of 90% keratin and thus are used as an organic fertilizer because they are good sources of amino acids, peptides, and minerals. Bacteria can degrade keratin through keratinase enzymes. These serine-type proteases have been used as alternatives to develop cost-effective, readily available, and eco-friendly nitrogen- and mineral-rich sources as organic fertilizers (Mazotto et al., 2010; Tamreihao et al., 2018).

Protease and protease-containing formulations can be used to clean hairs from clogged pipes and drains and can be used for depilation (Naveed et al., 2021).

Major contaminants in food bioprocessing sectors (e.g., milk and meat processing activities) result from protein-based residues. Alkaline protease has been used for waste management in different food-processing businesses as well as for activities at in residential areas (Majumder et al., 2015).

To minimize cleaning expenses, reduce environmental dangers, and increase equipment lifetime, various cleaning procedures have used protease alternatives. Because proteases are biodegradable, they will not cause environmental damage after they are used. Unlike other remediation approaches, biomass and chemicals cannot be removed to prevent accumulation. One disadvantage of using proteases for bioremediation is that the enzymes are expensive.

### 4.5. Special applications

#### 4.5.1. Contact lens cleaning

Cleaning solutions for contact lenses are often prepared using animal (e.g., pancreatin, trypsin, and chymotrypsin) and plant (e.g., papain) proteases. Most of these solutions cause the cleansing bath to exhibit an unpleasant smell or develop an odor after use for a few hours (Liu et al., 2018). Reportedly, however, some microbial proteases can clean the debris off of contact lenses and tear film, making these cleaning compositions odorless and safe. For example, proteases from *Bacillus* species, *Streptomyces* sp., and *Aspergillus* sp. do not cause allergic reactions or eye irritation (Singh and Bajaj, 2017; Razzaq et al., 2019; Rejisha and Murugan, 2021).

#### 4.5.2. Biotechnology processes

Some proteases are used for cleavage of various fusion tags after protein fusion expression in biotechnology protocols.

##### 4.5.2.1. SUMO Protease

Small ubiquitin-related modifier (SUMO) is a kind of ubiquitin-related protein that can be fused with the target protein to promote its solubility and enhance its soluble expression. After expression, SUMO protease can specifically recognize and cut the SUMO sequence from the target protein.

##### 4.5.2.2. Recombinant Kex2 protease

Kex2 protease, a yeast-derived precursor processing protease, is a calcium-dependent serine protease that specifically recognizes and cleaves the carboxy-terminal peptide bond of Arg-Arg, Lys-Arg, Pro-Arg and other bibasic amino acids. Kex2 protease was used for the cleavage of secreted peptides in yeast exogenous protein expression.

##### 4.5.2.3. TEV Protease

TEV protease is a cysteine protease of tobacco etch virus that specifically recognizes the heptapeptide sequence Glu-Asn-Leu-Tyr-Phe-Gln-Gly/Ser and cleaves between Gln and Gly/Ser amino acid residues and is commonly used as a protease to remove GST, HIS or other tags from fusion proteins.

##### 4.5.2.4. Proteinase K

This protease is used in genomic DNA extraction, enzyme digestion and removal in various common molecular biology experiments and cell biology experiments.

##### 4.5.2.5. Recombinant trypsin

This protease is an endopeptidase that can be used for the hydrolysis of C-terminal peptide bonds of lysine and arginine to split macromolecular proteins into small peptides. Trypsin is widely used in various biotechnological processes, such as cell separation of various tissues in cell culture experiments, degradation of denatured protein, enzymatic hydrolysis and sequencing of proteins, stem cell therapy, and cell therapy of tumors.

The sources of microbial proteases are extensive and may originate from any type of microorganism. Fungal proteases have been used in the food industry due to their safety and enzymatic characteristics. Acid proteases, among other functions, may be used as a substitute for activities associated with renin, papain, and pepsin. Species of *Aspergillus* and *Mucor* are important acid protease sources. Acid proteases from *Aspergillus flavus*, *Aspergillus oryzae*, *Aspergillus niger*, *Rhizomucor pusillus*, *Rhizomucor miehei*, and *Rhizopus* species are all used to prepare oriental foods, such as tempeh and koji, and to produce cheese as a substitute for rennet. Important milk clotting enzymes are found in *Rhizomucor pusillus*, *Rhizomucor miehei*, *Penicillium roqueforti*, *Penicillium camemberti*, and *Endothia parasitica* (Mandujano-González et al., 2016; Aleksandrina, 2021; Ha et al., 2022). Mesophilic fungi have been used to release proteolytic enzymes. Thermophilic fungi with good protease activity include *Achaetomium*, *Chaetomium*, *Humicola*, *Rhizomucor*, *Malbranchea*, *Penicillium*, *Rhizopus*, *Sporotrichum*, *Torula*, and *Talaromyces* (Johri et al., 1985). Many of these species produce sufficient levels of acid, neutral, alkaline proteases and milk-clotting enzymes. Thermophilic fungi offer low fermenter contamination at high growth temperatures, which is a selective advantage. Thermophilic molds exhibit better enzymatic abilities because of their greater production and higher thermostability, resulting in their widespread commercial applications. Their enzymatic reactions have specificity, rapid speed, and efficiency even in small quantities. The possibility of commercial isolation of some of these thermophilic fungal species has received increased attention (Macchione et al., 2007). Proteases are ideal candidates for laundry detergent because of their thermostability and activity at high pH (Gupta et al., 2002). Proteases from the genus *Bacillus* meet this requirement most often, so proteases used in laundry detergent mostly originate from the genus *Bacillus* (David et al., 2018; Rekik et al., 2019; Akram et al., 2020; Emran et al., 2020). Some proteases derived from extreme environmental microorganisms also frequently appear in this field (Abdullah et al., 2022).

In the past few decades, the application field of microbial proteases has rapidly expanded and has played an indispensable role in the food and detergent industries. With the continuous discovery of new microbial proteases, the application fields of microbial proteases will continue to expand, and their application methods will develop toward green and energy-saving directions.

## 5. Heterologous expression of proteases

Almost all microorganisms can generate proteases, but the amount of protease naturally generated by microorganisms may be very low, and isolating proteases is difficult. To obtain a certain number of proteases for conducting further research or application, heterologous expression of proteases is an important method; furthermore, with the development of bioinformatics, tens of millions of genes have been predicted as proteases, which is a very large potential resource pool of proteases. To obtain valuable proteases, only heterologous expression method can be used to produce enzyme proteins and then perform functional verification. In addition, some characteristics of natural proteases may not meet the requirements of industrial application, so it is necessary to perform modifications, and performing modification on the original enzyme through the heterologous expression system is convenient.

Heterologous expression systems can be divided into prokaryotic expression systems and eukaryotic expression systems. The most representative prokaryotic expression systems are the *Escherichia coli* expression system and *Bacillus subtilis* expression system. Eukaryotic expression systems are representative of *Pichia pastoris* expression systems, and they are the most commonly selected expression systems for protease heterologous expression. The above heterologous expression systems have the characteristics of high expression of target proteins, low expression of background proteins, high expression efficiency and easy operation (Demain and Vaishnav, 2009).

A steady stream of protease-encoding genes has been cloned and expressed in new hosts, and the three major organisms of choice for cloning and overexpression are *Escherichia coli*, *B. subtilis* and the *Pichia pastoris* expression system (Table 7).

**Table 7:** Heterologous expression of proteases.

Host strains for cloning and overexpression	Proteases or encoding genes	Plasmid vectors	References
B. subtilis			
*B. subtilis*	Glutamyl endopeptidase from *Bacillus intermedius* (gseBi)	pV	Shagimardanova et al. (2007)
*B. subtilis* DB104	A thermophilic neutral protease from *Bacillus stearothermophilus*	Shuttle vector pHP13	Zhang M. et al. (2008)
*B. subtilis*	An alkaline serine protease gene (GsProS8) from *Geobacillus stearothermophilus*	pWB980	Chang et al. (2021)
*B. subtilis* WB800	A gene coding for the nattokinase (Nk) from *B. subtilis* strain VTCC-DVN-12-01	pAC7	Nguyen et al. (2013)
*B.* subtilis WB800	A subtilisin-like alkaline serine protease (ASP) from *Bacillus halodurans* C-125	pMA0911	Tekin et al. (2020)
*B. s*ubtilis WB600	Keratinase (kerT) gene	pLY	Cao et al. (2019)
*B. subtilis* WB600	Alkaline serine protease (BcaPRO) from *B. clausii*	pWBPRO1 constructed based on pWB980	Liu et al. (2019)
*B. subtilis* SCK6	A novel streptomyces trypsin	pWB980	Wang et al. (2019)
A recombinant *B. subtilis*	Extracellular thermostable alkaline halophilic protease	pSaltExSePR5	Promchai et al. (2018)
*B. subtilis*	Alanine aminopeptidase from *Bacillus licheniformis* E7	pMA0911	Chen Y. et al. (2022)
*B. subtilis* SCK6	An extracellular keratinase	pMA0911	Tian et al. (2019)
A recombinant *B. subtilis*	Serine protease from *Bacillus intermedius*	pCB22	Sharipova et al. (2008)
*B. subtilis*	A fibrinolytic enzyme (subtilisin DFE) gene	pSUGV4	Peng et al. (2004)
*B. subtilis* WB600	Fibrinolytic protease of *Bacillus licheniformis* CH 3–17	pHY300PLK	Jo et al. (2011)
E. coli			
*E. coli*	An intracellular serine protease from isolated salt-tolerant *Bacillus* sp. LCB10	pET-30a	Hou et al. (2019)
*E. coli* Transetta (DE3)	Subtilisin-like protease from a thermophilic *Thermus thermophilus* HB8	pET-22b (+)	Xie et al. (2019)
*E. coli* strain BL21	Serine alkaline protease	pET-15b	Suberu et al. (2019)
*E. coli* BL21-Gold (Stratagene), *E. coli* ORIGAMI B and *E. coli* Rosetta2	Extracellular serine proteases from *Stenotrophomonas maltophilia*	pMS470Δ8	Ribitsch et al. (2012)
*E. coli* BL21(DE3) pLysS and *E. coli* BL21-AI™	Serine alkaline protease (SAPN) from *Melghiribacillus* genus.	pUT57 and pTrc99A, Gateway™ pDEST™ 17	Mechri et al. (2021)
*E. coli* BL21	A novel alkaline serine protease gene from native Iranian *Bacillus* sp.	pET-28 a (+)	Ariyaei et al. (2019)
*E. coli*	Serine protease from *Nocardiopsis* sp.	pET-39b (+) and pET-22b (+)	Rohamare et al. (2015)
*E. coli* BL21 (DE3)	A thermo- and surfactant-stable protease from *Thermomonospora curvata*	pET-25b (+)	Sittipol et al. (2019)
*E. coli* BL21 (DE3)	Serine proteases from *Oceanobacillus iheyensis* O.M.A18 and *Haloalkaliphilic* bacterium O.M.E12	pET-21a (+)	Purohit and Singh (2014)
*E. coli* BL21 (DE3)	Subtilisin-like Protease Myroicolsin	pET-22b (+)	Ran et al. (2014)
*E. coli* BL21	An alkaline protease from *Bacillus licheniformis*	pET–28b (+)	Lin et al. (2015)
*E. coli* BL21(DE3)	A fibrinolytic protease gene from the polychaeta, *Periserrula leucophryna*	pT7-7	Joo et al. (2013)
*E. coli* BL21(DE3)	A novel fibrinolytic serine protease from *Arenicola cristata*	pET-21a (+)	Zhao and Ju (2014)
*E. coli* BL21(DE3)	A novel aspartic protease gene from marine-derived *Metschnikowia reukaufii*	pET-24a (+)	Li et al. (2008)
*E. coli* BL21(DE3)	A novel extracellular subtilisin-like protease from the hyperthermophile *Aeropyrum pernix* K1	pGEX	Catara et al. (2003)
*E. coli* BL21(DE3)	A new thiol-dependent, alkaline serine protease	pET-28a (+)	Masilamani and Natarajan (2015)
*E. coli* BL21(DE3) pLysS	A serine protease-like protein from silkworm (*Bombyx mori*)	pGEX-5X-1	Kim et al. (2009)
*E. coli* BL21	Lon protease from rice (*Oryza sativa*)	pET-32a	Su et al. (2006)
*E. coli* Rosetta-gami (DE3)	Serine protease aprv2 from virulent isolate *Dichelobacter nodosus*	pET-22b (+)	Wani et al. (2016)
*E. coli* BL21 (DE3)	A novel extracellular cold-adapted alkaline protease gene of the marine bacterium strain YS-80-122	pET-28a	Wang et al. (2010)
*E. coli* Shuffle®T7.	Three main serine carboxypeptidases (SCP3, SCP20, and SCP47) from *Nepenthes mirabilis*	pET-28a-SUMO	Porfírio et al. (2022)
*E. coli* BL21(DE3)	Carboxypeptidases genes (dacA, dacB, dacC, and dacF) in *Bacillus subtilis* CW14	pET-28a (+)	Xu et al. (2021)
*E. coli*	A collagenolytic aspartic protease from *Thermomucor indicae-seudaticae*	pET-28a (+)	Pereira et al. (2020)
*E. coli* BL21(DE3) pLysS	Novel protease from *Bacillus licheniformis* strain K7A	pGEM-T Easy	Hadjidj et al. (2018)
*E. coli* BL21 (DE3)	A low salt-adapted extracellular protease from the extremely halophilic archaeon *Halococcus salifodinae*	pET-28a	Hou et al. (2021)
*E. coli* BL21	Avihepatovirus 3C protease	pET-32a	Sun et al. (2019)
*E. coli* BL21 (DE3) pLysS	Site-2 protease	pHEN6	Schacherl et al. (2017)
*E. coli* BL21 (DE3)	A dual-functional aminopeptidase from *Streptomyces canus* T20	pET-24a (+)	Qin et al. (2021)
P. pastoris			
*P. pastoris* SMD1168 and X33	Serine alkaline protease from *Melghiribacillus genus*	pPICZαC	Mechri et al. (2021)
*P. pastoris* GS115	Serine protease from thermophilic fungus *Thermoascus aurantiacus* var. levisporus	pPIC9K	Li et al. (2011)
*Saccharomyces cerevisiae* W3124	An aspartic protease	pYES 2.0	Bang et al. (1999)
*P. pastoris*	Proteinase K	pPink-HC	Skowron et al. (2020)
*P. pastoris* KM71	Protease from *Aspergillus niger*	pPIC9K	Ke et al. (2019)
*P. pastoris*	A plant aspartic protease (preprogaline B)	pGAPZα A	Feijoo-Siota et al. (2018)
*P. pastoris*	A novel serine protease	A self-construct plasmid	Shu et al. (2016)
*P. pastoris* X-33 and GS115 (his4)	Streptokinase	pPICZαA and pGAPZαA	Adivitiya Dagar et al. (2016)
*P. pastoris*	*Bacillus pumilus* 3–19 protease	pPINK-HC	Pudova et al. (2021)
*P. pastoris*	A zinc-dependent proteases of the metzincin superfamily of metalloproteases	pPIC9K	Schlenzig and Schilling (2017)
*P. pastoris* GS115	Recombinant protease MarP from *Mycobacterium tuberculosis*	pPICZα	García-González et al. (2021)
*P. pastoris*	A collagenolytic aspartic protease from *Thermomucor indicae-seudaticae*	pPICZαA	Pereira et al. (2020)
*P. pastoris*	A novel aminopeptidase B from *Aspergillus niger*	pPIC9K	Song and Feng (2021)
*P. pastoris* X-33 and SMD1168 strains	A *Mucor circinelloides* aspartic protease	pGAM1	Kangwa et al. (2018)
*P. pastoris*	A new serine protease from *Crotalus durissus collilineatus* venom	PICZαA	Boldrini-França et al. (2015)
*P. pastoris*	Keratinolytic serine protease gene sfp2 from *Streptomyces fradiae* var. k11	pPIC9K	Li et al. (2007)
*P. pastoris* KM71	Subtilisin Pr1A gene from a strain of locust specific fungus, *Metarhizium anisopliae*	pPIC9K	Zhang W. et al. (2008)
*P. pastoris* GS115	Subtilisin QK	pPICZα	Zhou et al. (2017)
*P. pastoris* SMD1168 and X33	A serine alkaline protease from *Melghiribacillus thermohalophilus*	pPICZαC	Mechri et al. (2021)
*P. pastoris* KM71	*Aspergillus sojae* alkaline protease	pPIC9K	Ke et al. (2018)
*P. pastoris* GS115	Recombinant *Aspergillus oryzae* alkaline protease	pPIC9K	Guo and Ma (2008)
*P. pastoris*	Keratinase (kerA) gene from *Bacillus licheniformis*	pPICZαA	Porres et al. (2002)
*P. pastoris*	A thermostable serine protease (TfpA) from *Thermomonospora fusca* YX	pPICZαA	Kim and Lei (2005)
*P. pastoris* GS115	The *Bacillus subtilis* subsp. subtilis str. BSP1 YwaD aminopeptidase	pHBM905A	Tang et al. (2016)
*P. pastoris* GS115	Carboxypeptidase Y from *Saccharomyces cerevisiae*	pHBM905A	Yu et al. (2014)
*P. pastoris* X-33	Prolyl aminopeptidase	pPIC9K	Yang et al. (2016)
*P. pastoris* X-33	X-Prolyl-dipeptidyl aminopeptidase from *Basidiomycete ustilago maydis*	pPICZαB	Juárez-Montiel et al. (2014)
*P. pastoris* X33 or SMD1168H	A metalloprotease	pPICZαC	Schlenzig et al. (2015)
*P. pastoris* GS 115	A new dipeptidyl-peptidase isolated from *Aspergillus fumigatus*	pHIL-S1	Beauvais et al. (1997)
*P. pastoris* GS115	A metalloprotease	pPIC9K	Song et al. (2021b)
*P. pastoris* GS115	A new carboxypeptidase from *Aspergillus niger*	pPIC9K	Song et al. (2021a)
*P. pastoris* GS115	Two new *Aspergillus niger* aspartic proteases	pPIC9K	Song Y. et al. (2020)
*Pichia pastoris* KM71H	Serine protease from *Bothrops pauloensis* snake venom	pPICZαA	Isabel et al. (2016)
*P. pastoris* GS115	A thermolysin-like protease, neutral protease I, from *Aspergillus oryzae*	pHBM905BDM	Ma et al. (2016)
*P. pastoris* GS115	First fibrinolytic enzyme from mushroom (*Cordyceps militaris*)	pPIC9K	Katrolia et al. (2019)
*P. pastoris* GS115	Thrombolytic enzyme (lumbrokinase) from earthworm	pPICZα-B	Ge et al. (2005)

From the above table, we can find that proteases from animals, plants, microorganisms or viruses can be successfully expressed in the three heterologous expression systems (*E. coli*, *B. subtilis* and *P. pastoris*). Another important feature of proteases is that a significant part is found in nature as a protein precursor (or zymogen). These proteases can be synthesized as inactive or less active precursor molecules, which have developed after evolution. These principal mechanisms can control the activity of proteases. The propeptide sequence of a protein precursor is connected to the C- or N-terminus of the material protein. The propeptides within protease precursors likely perform the following physiological functions: (1) help fold the mature enzyme; (2) provide the protease interaction with the bacterial cell surveillance mechanisms, including protease translocation through the cell wall; and (3) inhibit the proteases to protect the host cells from proteolytic damage (Baker et al., 1992; Baardsnes et al., 1998; Serkina et al., 2001; Varón et al., 2006).

For a protease to function, the propeptide must be removed, and the zymogen must be activated to produce a functional mature protease, so activation of the zymogen is important for proteases. Regulation of proteolytic enzyme activity is necessary for cells and tissues because proteolysis at the wrong time and location may be lethal.

The mechanisms by which zymogens activate proteolytic enzymes are diverse and naturally occurring. They are activated, in some cases, upon enzymatic or nonenzymatic cofactor triggering, an appropriate signal such as acidification, Ca^++^-binding or, in other cases, by limited intra- or intermolecular proteolysis cleaving off an inhibitory peptide (Khan and James, 1998; Marie-Claire et al., 1998; Takagi et al., 2001; Wiederanders et al., 2003).

However, regarding the heterologous expression of some proteases, their activation mechanism or whether the activation mechanism of zymogens occurs in the heterologous host are unable to predict in advance; whether the propeptide is retained is unknown? Some relevant reports were summarized and the result showed that most of the successfully expressed proteases were expressed with the retention of the propeptide, but some of them were successful in removing the leading peptide and expressing the mature peptide directly (Table 8).

**Table 8:** Heterologously expressed proteases with (without) the retention of the propeptide.

Proteases	Protease gene sources	Expression host	Expression with propreptide (Y/N)	Remarks	Reference
A keratinolytic serine protease	*Streptomyces fradiae* var.k11	*E. coli*	Y	Recombinants expressing the proenzyme exhibited markedly higher activity than that recombinant expressing mature enzyme	Meng et al. (2007)
Keratinase	*Bacillus licheniformis*	*Pichia pastoris*	Y	Expressing the pro-mature structure was reported to increase the production of the keratinases gene (kerA)	Carlsson et al. (2001)
A thermolysin-like neutral protease	*Bacillus stearothermophilus*	*E. coli*	N	A much more effective access to active mature protease was found when TLP-ste (devoid of its prosequence) was expressed this confirming that the propeptide is not essential for proper folding of the enzyme or its stabilization during the folding process	Mansfeld et al. (2005)
A novel extracellular serine protease	*Aeropyrum pernix* K1	*E. coli*	N	/	Catara et al. (2003)
Subtilisin	*Thermus thermophilus* HB8	*E. coli*	Y	Expression of the mature-subtilisin gene was found to produce inactive inclusion bodies, expression of the pro-subtilisin gene resulted in active mature-subtilisin	Xie et al. (2019)
Subtilisin E	*Bacillus subtilis* 168	*E. coli*	Y	When the entire coding region for pre-pro-subtilisin E was cloned into an *Escherichia coli* expression vector, active mature subtilisin E was secreted into the periplasmic space; When the propeptide was absence to the mature subtilisin sequence, no protease activity was detected	Ikemura et al. (1987)
*Candida* secreted aspartic proteases	*Candida albicans*	*Pichia patoris*	N	Expression of the *C. albicans* SAP1 gene lacking the propeptide-coding region in the *Pichia pastoris* does not lead to the secretion of the enzyme into the culture supernatant, but results in an accumulation of recombinant protein in the cell. Co-expression in this system of the unattached propeptide from Sap1p, as well as from other Saps, restored Sap1p secretion	Beggah et al. (2000)
Keratinase	*Bacillus licheniformis* BBE11-1	*E. coli*	Y	Optimizing the C-terminus of propeptide will affect the cleavage efficiency of propeptide. The primary structure of C-terminus propeptide is crucial for the mature keratinase production.	Liu et al. (2014)
Leucyl aminopeptidase A	*Aspergillus oryzae* RIB40	*E. coli* and *Pichia pastoris*	Y	/	Baltulionis et al. (2021)
Subtilisin-like serine proteases	Tomato	*E. coli*	Y	/	Meyer et al. (2016)
Nattokinase	*Bacillus subtilis* VTCC-DVN-12-01	*Bacillus subtilis* WB800	Y	/	Nguyen et al. (2013)

For the heterologous expression of a novel protease, the precursor sequence should be cloned, and if there is no functional enzyme expression, then in turn clone the mature peptide sequence. The identification of the propeptide sequence of a protease can be performed by referring to the literature or using prediction software of the peptide clearance sites: https://services.healthtech.dtu.dk/service.php?ProP-1.0. Notably, the propeptide sequence of a protease may be at the N-terminal end or the C-terminal end of the protein sequence.

Heterologous expression is an important method for detecting novel microbial proteases and will continue to play a crucial role. Moreover, as an optimization of this approach, some new technologies, such as CRISPR and directed evolution, will continue to be applied to optimize this approach and improve the method’s efficiency. As representatives of prokaryotic expression systems, *E. coli* expression systems and *B. subtilis* expression systems, as well as eukaryotic expression systems, *P. pastoris* expression systems can now meet the heterologous expression of proteases from prokaryotic and eukaryotic microorganisms. Expression levels could be increased and more functional protease expression could be obtained to further improve heterologous expression.

## 6. Conclusion and future prospects

Because enzymes are environmentally friendly chemicals, they could completely replace or reduce the use of hazardous chemicals in industrial processes. As a result, enzymes show promising applications for sustainable manufacturing and production. Proteases are superior to many industrial enzymes because of their varied application in many different bioindustries, such as detergent, leather, textiles, and food, as well as pharmaceutical, biotechnology, and waste treatment processes. Among proteases from diverse sources, microbial proteases have been the preferred source for applications owing to their fast growth, efficient production, wide diversity, longer shelf life, and potential for genetic manipulation of microorganisms compared to plant or animal sources.

It is certain that microbial protease, as a green, efficient tool, will be continuously applied in various industry applications with the development of biological technology, and it will lead the development of the abovementioned fields or promote the development of each field. To increase our fundamental knowledge on microbial ecology (e.g., enzymes, their evolution, and their relevance in industrial sectors), “omics” and biological technologies should continue to be used for molecular characterization, crystallography, and enzymatic modulation by applying algorithms, bioinformatics tools, and genetic engineering. Genetic engineering and immobilization techniques should be further developed to discover new proteases, enzyme systems that are more effective and efficient should be developed, the functions of existing proteases should be optimized, and fewer resources and energy should be consumed while achieving maximum product yields.

In the next decade or few decades, research should be conducted on proteases regarding enzyme preparation methods and usage methods to improve efficiency, such as developing immobilized enzyme technology, enzyme modification technology, and protease fusion application with chemical approaches and developing faster and more efficient methods for detecting and analyzing proteases to facilitate the development of new proteases. *De novo* design of new proteases using artificial intelligence and various algorithms should also be applied. It is also necessary to develop general methods for long-term preservation of proteases to mitigate inactivation caused by self-hydrolysis; greater accuracy and control during the production process is critical in terms of improving product value or expanded substrate extensiveness of proteases with a goal of obtaining better hydrolysis efficiency. Biochemical attributes of microbial proteases, such as thermostable, cold-active, and halophilic extreme environmental properties, should be further studied to determine significant applications in bioprocesses, as proteases and even enzymes are always of research interest.

## Author contributions

PS and FWe: conceptualization. PS, XZ, SW, WX, and FWa: literature search. PS, XZ, and FWa: writing–original draft preparation. PS, SW, FWe, and RF: writing–review and editing. PS and FWe: funding acquisition. All authors contributed to the article and approved the submitted version.

## Funding

This work was supported by the Natural Science Foundation of Shandong Province (grant number ZR2022MC159).

## Conflict of interest

PS and SW were employed by Shandong Aobo Biotech Co. Ltd.; PS and RF were employed by Jiangxi Zymerck Biotechnology Co., Ltd.

The remaining authors declare that the research was conducted in the absence of any commercial or financial relationships that could be construed as a potential conflict of interest.

## Publisher’s note

All claims expressed in this article are solely those of the authors and do not necessarily represent those of their affiliated organizations, or those of the publisher, the editors and the reviewers. Any product that may be evaluated in this article, or claim that may be made by its manufacturer, is not guaranteed or endorsed by the publisher.
